# Ensemble of Deep Learning Models for Sleep Apnea Detection: An Experimental Study

**DOI:** 10.3390/s21165425

**Published:** 2021-08-11

**Authors:** Debadyuti Mukherjee, Koustav Dhar, Friedhelm Schwenker, Ram Sarkar

**Affiliations:** 1 Department of Computer Science and Engineering, Jadavpur University, Kolkata 700032, India; debadyuti23@gmail.com (D.M.); koustavdhar.31.05@gmail.com (K.D.); ram.sarkar@jadavpuruniversity.in (R.S.); 2 Institute of Neural Information Processing, University of Ulm, 89069 Ulm, Germany

**Keywords:** sleep apnea, ECG signal, ensemble, deep learning, health monitoring

## Abstract

Sleep Apnea is a breathing disorder occurring during sleep. Older people suffer most from this disease. In-time diagnosis of apnea is needed which can be observed by the application of a proper health monitoring system. In this work, we focus on Obstructive Sleep Apnea (OSA) detection from the Electrocardiogram (ECG) signals obtained through the body sensors. Our work mainly consists of an experimental study of different ensemble techniques applied on three deep learning models—two Convolutional Neural Network (CNN) based models, and a combination of CNN and Long Short-Term Memory (LSTM) models, which were previously proposed in the OSA detection domain. We have chosen four ensemble techniques—majority voting, sum rule and Choquet integral based fuzzy fusion and trainable ensemble using Multi-Layer Perceptron (MLP) for our case study. All the experiments are conducted on the benchmark PhysioNet Apnea-ECG Database. Finally, we have achieved highest OSA detection accuracy of 85.58% using the MLP based ensemble approach. Our best result is also able to surpass many of state-of-the-art methods.

## 1. Introduction

In this modern era, the role of health monitoring systems is increasing in our daily life. Older people are the most benefited ones from the merits of monitoring their health. Smart sensors, under any healthcare system attached to various body parts, can sense and record the required features of the human body. These kinds of sensors can be placed in any smart-watch or smartphone. The introduction of the Internet of Things (IoT) in the healthcare domain has further upgraded the facilities [1]. Health-based alarms, personal smart medical recommendations, etc. have decreased the life-risks caused by sudden health problems.

One such problem under the domain of health monitoring is sleep apnea detection. Sleep Apnea HypoApnea Syndrome (SAHS) or simply apnea is a common sleep disorder related to interruption in breathing during sleep. In most cases, older people are affected by this syndrome [2]. OSA is a category of apnea that causes partial or complete blockage of the airway in our body. OSA may further cause sleepiness, fatigue, morning headache, etc. [3]. Statistics say that almost 9% of the men and 4% of women among the middle-aged people suffer from the mentioned sleep disorder. The sleep loss caused by OSA may lead to some long-term diseases like cardiovascular diseases [4]. Thus, a smart health monitoring system is beneficial for the diagnosis of apnea so that early measures can be taken.

The most common method for apnea diagnosis is ECG [5]. ECG records the electronic signals generated from the human heart. It serves the purpose to detect whether our heart is abnormally working or not. In this work, we have divided the whole time-series ECG data into time-intervals of equal length. Then we have used CNN [6] based deep learning models along with ensemble learning to detect apnea in the given time-span. We have chosen three previously proposed CNN models as base models: (i) CNN architecture proposed by Wang et al. [7], (ii) CNN model proposed by Sharan et al. [8], and (iii) combination of CNN and LSTM network [9] proposed by Almutairi et al. [10]. To aggregate the base models’ predictions and to yield better results, we have applied four ensemble approaches: (i) Majority Voting, (ii) Sum rule, (iii) Choquet Integral based fuzzy fusion and (iv) Trainable ensemble using MLP. Our work involves the experimental study between these four ensemble techniques.

The main advantage of ensemble learning is that it considers and combines all the decisions by different models rather than relying on a single classifier [11]. An ensemble will be successful if its component classifiers have diversity while making the prediction. Also, the ensemble formation will not serve any purpose if all the components generate too many inaccurate predictions [11].

**Figure 1:**
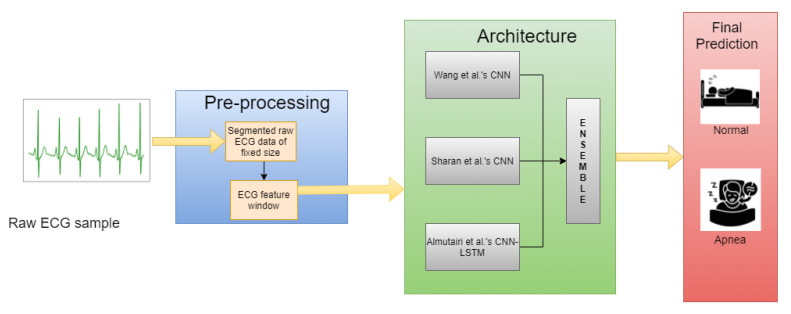
Overall workflow of the ensemble based OSA detection model.

We have chosen the PhysioNet Apnea-ECG Database [12], a standard and publicly available dataset, to conduct all the required experiments. To summarize, first, we form segments from the raw ECG data from the benchmark database, perform necessary pre-processing to derive important features and then train the three base models. Next, all three deep learning models predict the test data and the final prediction is generated by applying the ensemble technique of choice. Figure 1 pictorially represents the above-mentioned process. The rest of the work consists of the four sections, namely, Related Work, Materials and Methods, Results and Discussion, and Conclusions.

## 2. Related Work

Since OSA or any other kind of apnea detection is a classification problem of the two classes—normal and apnea, machine learning classifiers like Support Vector Machine (SVM) [13], k-Nearest Neighbours (kNN) [14], Random Forest (RF) [15] etc., and deep learning classifiers like CNN etc., are very much applicable in this domain. Like any other clinical diagnosis, detection of sleep apnea has become an important research topic in the healthcare domain.

Ng et al. [16] have used thoracic and abdominal signals as input features for sleep apnea indication and have achieved 70.29–86.25% sensitivity. Alvarez et al. [17] have worked on the non-linear analysis of blood oxygen saturation (Sa) obtained from nocturnal oximetry. From the experiments, they have discovered 111 out of 187 subjects as OSA positive. Qin et al. [18] have studied the effect of OSA in Heart Rate Variability (HRV). They have conducted the experiments on 426 normal and 826 OSA affected subjects and have discovered that HRV tends to reduce with the severity of apnea disease.

Although there are many statistical body measures like ECG, acoustic speech signal, Sa, Electroencephalogram (EEG) available for apnea diagnosis [5], we have solely focused on ECG signal for our work. A lot of research works on apnea diagnosis from ECG signals have already been performed. Almazaydeh et al. [5] have extracted the relevant statistical features such as mean, standard deviation, median, inter-quartile range and some of their derivations for an RR interval (interval between two consecutive R peaks) of the raw ECG signals of the PhysioNet Apnea-ECG database [12]. They have applied SVM on these extracted features and have achieved a maximum of 96.5% accuracy. Cheng et al. [19] also have conducted experiments on RR intervals of the ECG signal of the PhysioNet Apnea-ECG database. By applying the Recurrent Neural Network (RNN) [20], they have achieved 97.80% accurate results.

Nguyen et al. [21] have considered the Recurrence Quantification Analysis (RQA) statistics of the HRV data of PhysioNet Apnea-ECG database as features. Initially, they have performed the classification task by using both SVM and Artificial Neural Network (ANN). They have used soft decision fusion to aggregate both the classifiers’ scores and have obtained 85.26% accurate results. Hassan et al. [22] have pre-processed the raw ECG signal of the PhysioNet Apnea-ECG database by applying the Tunable-Q factor Wavelet Transform (TQWT). They have used Adaptive Boosting (AdaBoost) [23], an ensemble method applicable to the decision tree and achieved 87.33% accurate results.

Wang et al. [24] have considered the past time-windows for training the MLP architecture. Such time-windows are restricted to have a time-span of a minute, whereas each sample under the respective time-span has the six time-domain RR Interval (RRI) features—MRR (mean of RRI), MHR (mean of heart rates), RMSSD (root mean square of differences between adjacent RRIs), SDNN (standard deviation of RRIs), NN50 (number of adjacent RRIs exceeding 50 milliseconds) and pNN50 (NN50 divided by the number of RR intervals) and six frequency domain R-peak Amplitude features—Very Low Frequency (VLF), Low Frequency (LF), High Frequency (HF), LF/(LF + HF), and HF/(LF + HF). Finally they have achieved the best result with 87.3% accuracy. Shen et al. [25] have proposed MultiScale Dilation Attention 1-D CNN (MSDA-1DCNN) for extracting features from the RRI and have applied Weighted-Loss Time-Dependent (WLTD) classification model for OSA detection and have achieved 89.4% accuracy on the PhysioNet Apnea-ECG database [12].

Chang et al. [26] have proposed a novel 1-D CNN architecture for the purpose of OSA detection. In their work, each one-minute segment of the raw ECG signal is initially undergone through the band pass filtering followed by Z-score normalization before fitted into the CNN model. Overall, they have achieved 87.9% accuracy on the PhysioNet Apnea-ECG database [12] whereas, the performance has increased up to 97.1% in the case of pre-recorded samples. Thompson et al. [27] have proposed a 1-D CNN architecture including a convolution layer, a max pooling layer, a fully connected MLP and a softmax output layer. In their work, they’ve applied a windowing strategy, with window sizes of 500, 1000, 1500, 2000 and 2500 for validation of their model, which achieved 93.77% accuracy for window size of 500 on the PhysioNet Apnea-ECG database [12]. Mashrur et al. [28] have proposed a novel Scalogram-based CNN to detect OSA using ECG signals. In their work, they’ve obtained hybrid scalograms from the ECG signals using continuous wavelet transform (CWT) and empirical mode decomposition (EMD). They train a CNN model on these scalograms to extract deep features to detect OSA, achieving an accuracy of 94.30% on the PhysioNet Apnea-ECG database [12].

The majority of the previous works have considered ECG, and this fact motivates us to choose ECG signal data for conducting our work. PhysioNet Apnea-ECG database is also a popular one for working on OSA detection. We have chosen deep learning models for our work as they are very much applicable to the time-series data [6]. However, only raw samples cannot produce outstanding results when fit into CNN models as discussed in the Results and Discussion section, hence it requires some pre-processing. Since our main concern is about the ensemble approaches in apnea detection domain, some of the established works based on ensemble techniques are also discussed.

Faußer et al. [29] have applied Temporal Difference (TD) and Residual-Gradient (RG) update methods on a given set of agents with their own nonlinear function approximator, for instance, an MLP to adapt the weights to learn from joint decisions, such as Majority Voting and Averaging of the state-values. Also, Glodek et al. [30] have worked on ensemble approaches for density estimation using Gaussian Mixture Models (GMMs) by combining individual mixture models incorporating a high diversity to create a more stable and accurate model. Chakraborty et al. [31] have performed ensemble of filter methods, such as optimal subsets of features using filter methods Mutual Information (MI), Chi-square, and Anova F-Test, and with the selected features building learning models using MLP based classifier.

Kächele et al. [32] have used an ensemble of RF, Radial Basis Function (RBF) networks to determine the intensity of pain based on the video shown and body features such as ECG, Electromyography (EMG). They have used MLP to train from the classification scores obtained from individual base models for score level fusion. Dey et al. [33] have used a weighted ensemble of three CNN based models: ADNet, IRCNN and DnCNN to remove white Gaussian noise from an image. The aforementioned three models’ outputs are aggregated in the ratio 2:3:6 respectively. Bellmann et al. [34] have applied various fusion approaches for the Multi-Classifier System (MCS) to effectively measure pain intensity levels. Their case study includes one of the most popular fusion techniques—bagging and boosting. Kundu et al. [35] have proposed a fuzzy-rank based classifier fusion based approach which uses the Gompartz function for determining the fuzzy-ranks of the base classifiers. They have conducted the experiments on the SARS-COV-2 [36] and Harvard Dataverse [37] datasets for diagnosing COVID-19 from the CT-scans and have achieved the best results with 98.93% and 98.80% accuracies respectively by using the ensemble of the pre-trained models VGG-11, Wide ResNet-50-2 and Inception v3.

All these previously established works prove that the application of ensemble is spread out through many research fields. The huge success and scope of research in classifier fusion are the main reasons for its popularity. Still based on our knowledge, any ensemble technique based work has not been conducted for apnea diagnosis till now.This has motivated us to conduct experimental studies base on ensemble techniques on the OSA detection domain. Additionally, we have chosen the three deep learning models—(i) Wang et al.’s [7] proposed CNN model, (ii) Sharan et al.’s [8] proposed CNN model, (iii) Almutairi et al.’s [10] proposed CNN-LSTM model as base models. The reason for such choice is these three models are all CNN based which are robust, excellent classifiers in general. The fact that the chosen three models have previously been used for OSA detection further encourages us to work with them. Thus, we have conducted our work by applying an ensemble of CNN based architectures in the popular PhysioNet Apnea-ECG database [12].

## 3. Materials and Methods

### 3.1. Datasets Used

We chose the PhysioNet Apnea-ECG database [12] for conducting all the experiments. A total of 70 records sampled at 100 Hz frequency were present in the database out of which 35 records were in the training set and the rest belonged to the testing set. People belonging to the 27–30 year age group volunteered in the data collection process. The data collection procedure lasted approximately 7–10 h per subject.

After the segmentation process, train data and test data had 15,961 and 15,938 pre-processed noiseless 1-min samples respectively. The training set had 9832 samples of apnea class and 6129 samples of normal class respectively, and the test set had 9838 samples of apnea class and 6100 samples of normal class respectively.

### 3.2. Methodology

#### 3.2.1. Pre-Processing

We performed the steps to convert the raw ECG signal data into 2-D matrices in this stage by following the pre-processing approach according to the GitHub link (https://github.com/zzklove3344/ApneaECGAnalysis, accessed on: 6 August 2021, accessed from: Kolkata, India). So, the pre-processing consists of the following steps:

- First, the raw data were transformed into segments having a time-span of 1 min. Thus, each segment had a size of 6000×1.
- We further divided each 1 min sample by dividing it into 240 parts so that each division contained 25 consecutive samples. Next, we extracted the features from each sub-division.
- We applied the Multilevel Teager Energy Operator (MTEO) algorithm by using the Matlab ToolBox BioSigKit to evaluate the R peaks, ECG Derived Respiration signal (EDR) from the raw segments. MTEO algorithm was used for action potential detection to locate the QRS complexes in Electromyography signals [38].
- From the R peaks, we further derived (i) RRI—the interval between two consecutive R peaks, (ii) R peak Amplitude (RAMP). Thus, three features were derived from each segment. Based on RRI, Each ECG segment is classified as (i) noise, (ii) clear.
- RRI and RAMP features went though the Smoothing and Spline interpolation processes. We also downsampled the EDR signal values.
- After all these processes, the clear segments were present as 2-D matrices having the size of 240×3.
- We normalized each feature by applying Z-score normalization. The formula of Z-score normalization was: $\hat{X}=\frac{\left(X-\mu_{X}\right)}{\sigma_{X}}$ where, *X* is the original feature column, $\hat{X}$ is the normalized feature column and $\mu_{X}$, $\sigma_{X}$ are the mean and standard deviation of the feature values in *X*.

To perform five-fold cross-validation, we combined the pre-processed training and test data, accumulating to a total of 31,899 samples. Then, the combined dataset underwent shuffling before splitting for the cross-validation. For the five-fold cross-validation, the overall dataset was divided into five parts where five parts were used for training and the remaining part is used for testing purposes, over all the combinations.

#### 3.2.2. Models Used

In this work, we used three existing CNN based models—(i) Wang et al.’s [7] proposed CNN architecture, (ii) Sharan et al.’s [8] proposed CNN model, (iii) Almutairi et al.’s [10] proposed CNN-LSTM model for making the initial predictions. These models have previously been proposed in this very domain. However, we made some minor changes to the original architecture.

**Figure 2:**
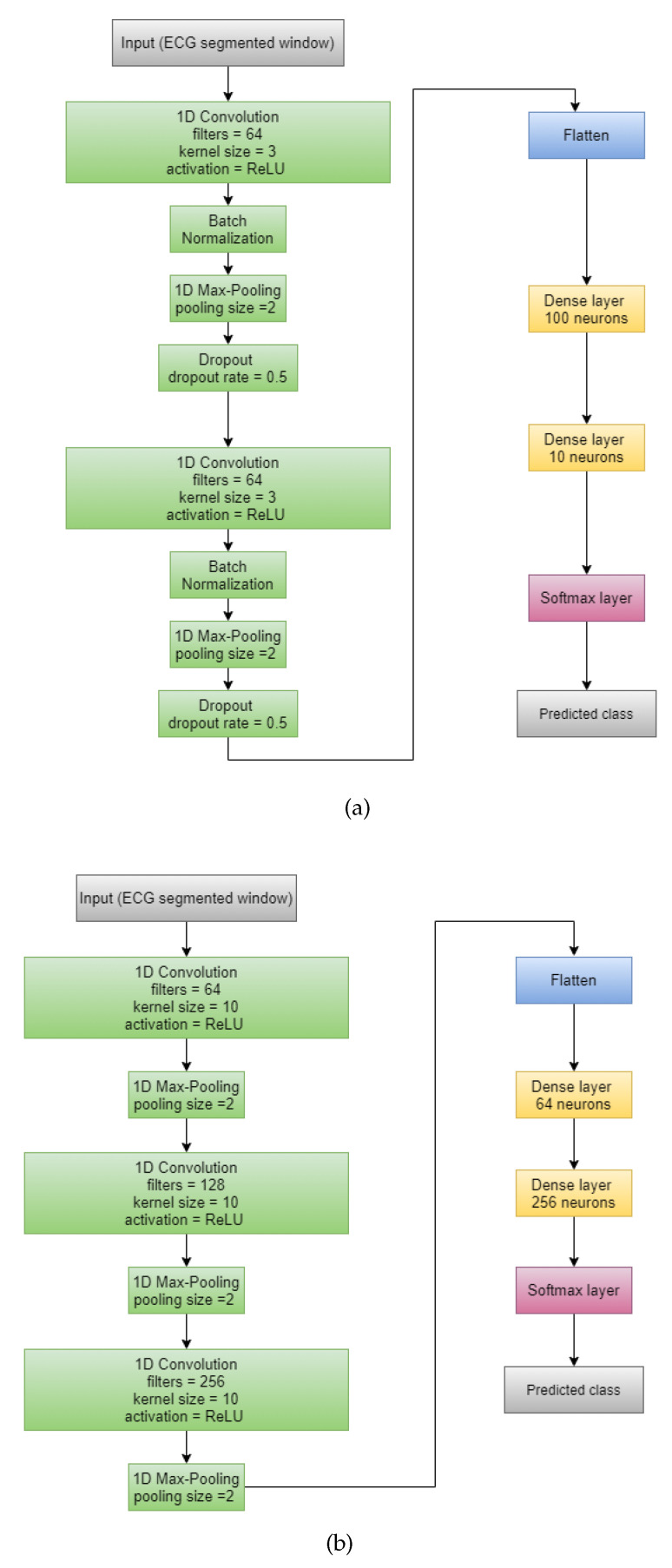
Architectures of the CNN models originally proposed by (**a**) Wang et al. [7] and (**b**) Sharan et al. [8] respectively.

**Figure 3:**
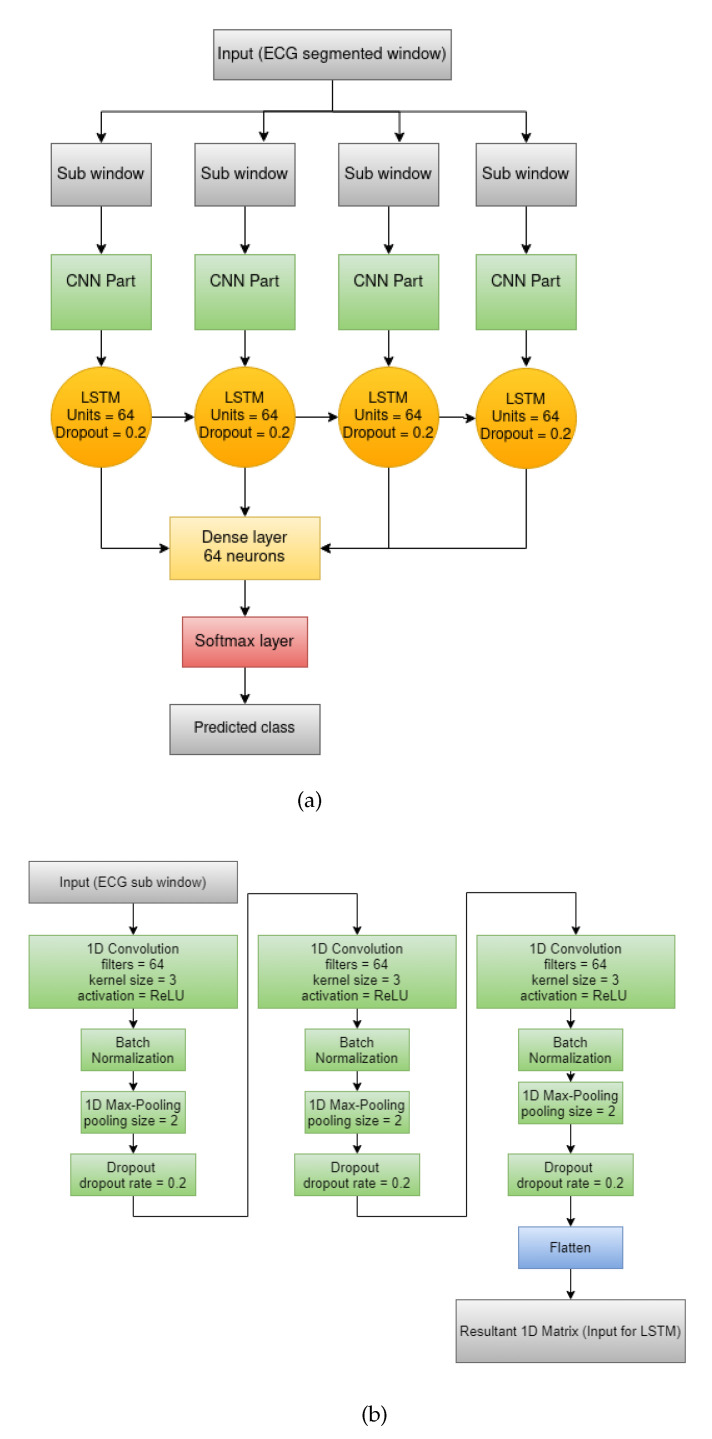
Almutairi et al.’s [10] proposed CNN-LSTM model: (**a**) overall architecture, (**b**) architecture of the CNN part present in the overall structure.

1. Wang et al.’s [7] CNN: We replaced all the 2-D layers of the original architecture with 1-D layers for the convenience of handling the time series ECG signal data. As we considered only three features for each sample, 2-D CNN models were not applicable on the pre-processed data. At first, we used two CNN blocks consisting of a 1-D Convolution layer with a kernel size of 3, 64 filters, Rectified Linear Unit (ReLU) as activation function followed by Batch-Normalization layer and a 1-D Max Pooling layer of size 2. Next, a Flatten layer followed by two Dense layers with 100 and 10 neurons were applied to the output produced by the final CNN block. Finally, the probability for each class was calculated using the Softmax layer. The architecture of this model is shown in Figure 2a.
2. Sharan et al.’s [8] CNN: The model consisted of three CNN blocks having a 1-D Convolution layer with kernel size 10 with ReLU as activation function followed by a 1-D Max Pooling layer with a pooling size of 2. Each of these three blocks differed in the number of filters in the 1-D Convolution layer (64, 128 and 256). Next, two Dense layers with 64 and 256 neurons were applied. Finally, the Softmax layer was used for calculating the probabilities of each class. Figure 2b shows the whole structure of this model.
3. Almutairi et al.’s [10] CNN-LSTM: The proposed model consisted of three 1D- Convolution blocks each having a 1D-Convolution layer with kernel size 3 with ReLU activation function followed by a Batch-Normalization layer, 1D-Max Pooling layer of pooling size = 2 and a Dropout layer having a dropout rate of 0.2. These three blocks differed only in the number of filters applied on the 1-D Convolution layer (64, 128 and 16). A Flattened layer followed by an LSTM layer having an output size of 64 was applied after the CNN blocks. Next, a Dense layer having 64 neurons was used. To be applicable for the CNN-LSTM architecture, we initially divided each window into four consecutive sub-windows and then have applied the CNN layers on each of the sub-windows. The LSTM layer worked on all four sub-windows collectively. The final output i.e., the probability for each class was given by the Softmax layer. We provided Figure 3a to represent the whole architecture of the proposed CNN-LSTM model whereas Figure 3b was provided to show the CNN-only part separately.

### 3.3. Applied Ensemble Methods

For the later parts of the experiment, we used different ensembles approaches to further increase the overall classification accuracy. Ensemble approach could be classified into two categories,

1. Decision level fusion: Here the predicted class i.e., only decision was considered for each model.
2. Score level fusion: Here the prediction score for each of the class was considered for each model.

We applied one decision level ensemble— Majority Voting and three score level ensemble procedures, namely Sum Rule, Choquet integral based fuzzy fusion and trainable MLP ensemble. Let us assume there are a total of *k* classes $c_{1},c_{2},\ldots c_{k}$ and *l* classifiers $m_{1},m_{2},\ldots m_{l}$. Model $m_{j}$ predicts for a sample *x* being in class $c_{i}$ with probability $p_{ij}$. So, the prediction class of that sample given by model $m_{j}$ will be $\hat{c_{j}}=argmax_{i}\left(p_{ij}\right)$.

**Figure 4:**
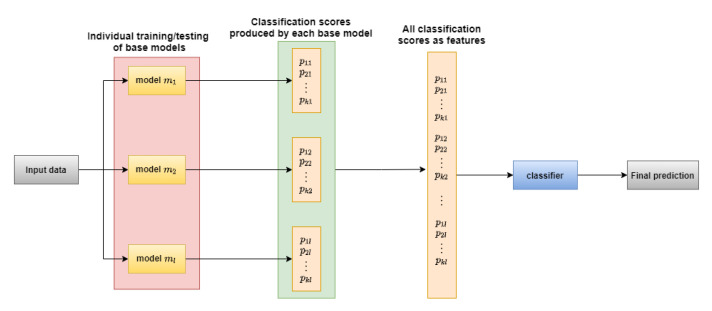
Basic architecture of a trainable ensemble model.

The aforementioned ensemble techniques could also be divided into the following two categories based on whether training was performed for ensemble learning or not:

1. Non-trainable ensemble: In this case, we applied some pre-defined rules to aggregate the decisions/scores made by all the base models. majority voting, Sum rule and Choquet integral based fuzzy fusion were all such ensemble approaches used in this work.
2. Trainable ensemble: In this case, we used another classifier to perform the aggregation of all base models’ scores. All class scores given by all the base models were flattened into a single feature vector sample-wise. Next, we chose an appropriate classifier that produced good results when train and test performed against the flattened train and test data scores. In our work, we chose an MLP for the aggregation task. Figure 4 represents the working procedure of a trainable ensemble method.

The following paragraphs contain a brief discussion about all of the ensemble approaches used in our work.

**Figure 5:**
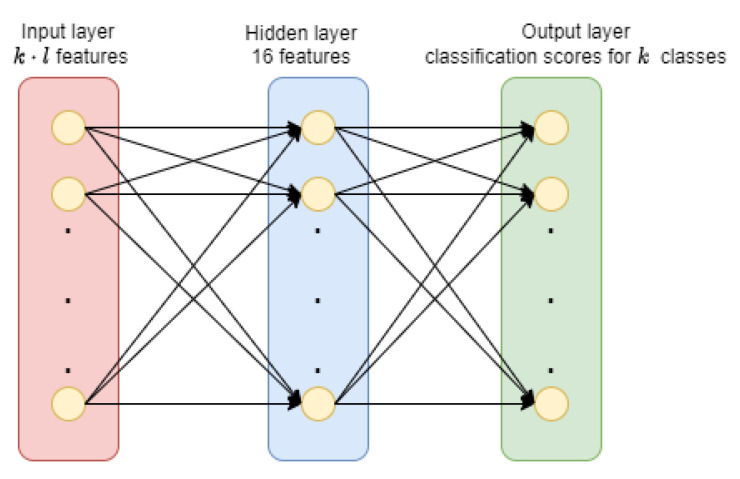
Architecture of MLP used for ensemble where *k* denotes total number of classes and *l* denotes total number of base-classifiers.

1. Majority voting: In this kind of ensemble, the class predicted by maximum number of models is assigned as the final prediction. So, the final class would be $argmax_{i}\left(freq\left(c_{i}\right)\right)$,
  where, $freq(c_{i})$ is the number of the number of models predicted class $c_{i}$.
2. Sum rule: The class with maximum sum of scores of all classifiers is the final predicted class. So the final prediction class would be $argmax_{i}\left(\sum_{j}p_{ij}\right)$.
3. Choquet integral based fuzzy fusion: Fuzzy integrals are generally aggregation operators which combine the information in this case, confidence values of all sources and their all possible combinations. Each source i.e., classifier has been given initial weights called fuzzy measures.
  Fuzzy measure of set *X* is a function g:P(X)⟶[0,1] where P(X) is the power-set of *X* which holds the following two conditions [39]:
  (a) The boundary of *g* should follow: g(ϕ)=0 and g(X)=1.
  (b) Suppose *A*, *B* are two subsets of *X* and A⊆B then the g(A)≤g(B) must be satisfied.
  In this case, the set *X* becomes the set of all classifiers i.e., $X=\{m_{1},m_{2},\ldots m_{l}\}$.
  Like Pacheco et al.’s [40] work, we used entropy of each classifier’s probability vector to evaluate the fuzzy measure. The formula of entropy of probability vector given by classifier $m_{j}$, $E_{j}$ is given as:
  (1) $$E_{j}=\sum_{i}p_{ij}\cdot log\left(p_{ij}\right)$$
  From the function in Equation (1), we understand that entropy becomes less when the probability for one class is much larger than that of other classes. So, it would be inverse to the goodness of a classifier. Hence, we used subtracted entropy value from the maximum possible entropy value (1 in this case) to get the fuzzy measure for each classifier. Finally, each of the fuzzy measure was divided by the sum of all fuzzy measure values. Suppose $g_{1},g_{2},\ldots g_{l}$ are the fuzzy-measures provided for the classifiers $m_{1},m_{2},\ldots m_{l}$ respectively which means-
  (2) $$g_{j}=\frac{1-E_{j}}{\sum_{i}\left(1-E_{i}\right)}$$
  where, $E_{j}$ is the entropy of the probability vector provided by classifier $m_{j}$ evaluated according to Equation (1).
  Tahani et al. [41] have introduced the concept of Sugeno λ-measure effective for fuzzy measures. It holds the additional property: if A∩B=ϕ then there exists that λ>−1 such that-
  (3) g(A∪B)=g(A)+g(B)−λ·g(A)·g(B)
  where, *A* and *B* are both subsets of *X*. Considering the additional property, λ can be evaluated as the solution for the equation:
  (4) $$\lambda +1=\prod_{j}\left(\lambda \cdot g\left(\left\{m_{j}\right\}\right)+1\right)$$
  where, $\left\{m_{j}\right\}$ is the singleton set containing $m_{j}$. So, after evaluating the fuzzy measures for all singleton sets, we have evaluated the fuzzy measure for the combination of classifiers using Sugeno λ-measure.
  The Choquet integral [42] is used for aggregating the scores based on all combination of classifiers. Based on the fuzzy measures, this integral can even combine the empirical strategies like addition, multiplication of scores produced by the classifiers. Choquet integral for class $c_{i}$ can be evaluated according to the following formula:
  (5) $$C_{g}\left(X\right)=\sum_{j}s_{\pi_{j}}\cdot \left[g\left(A_{\pi_{j}}\right)-g\left(A_{\pi_{j-1}}\right)\right]$$
  where, $s_{j}=p_{ij}\forall j=1,2,\ldots l$ and the set of classifiers *X* is permuted such that $s_{\pi_{1}}\geq s_{\pi_{2}}\geq \ldots \geq s_{\pi_{l}}$. $\left[g\left(A_{j}\right)-g\left(A_{j-1}\right)\right]$ depicts the relative importance of the classifier $m_{j}$.
  So all the class-scores $CS_{i}$ are obtained from Equation (5) and class with the maximum score will be the final predicted class which is $argmax_{i}\left(CS_{i}\right)$.
4. Trainable ensemble using MLP: The main building blocks of a Neural Network (NN) [43] are nodes. These nodes usually remain collectively as layers. Information in NN passes from layer to layer. In the Feed-forward Neural Network (FNN) [44], the flow of information is fixed in one direction, i.e., from the input layer to the output layer.
  Suppose there are n nodes in the previous layer where each node *i* forwards the value $x_{i}$ to a particular node *j* in the current layer. Then, the output *y* of the node *j* will be-
  (6) $$y=\phi (\sum_{i}\left(w_{i}\cdot x_{i}\right))$$
  where ϕ is the activation function present in the current layer and $w_{i}$ be the weight assigned to the path from node *i* to node *j*. The main objective of FNNs is to optimize these $w_{i}$s.
  In any MLP [45] architecture, the FNN must have at least one hidden layer between the input and the output layer. In our work, we considered a simple MLP architecture with only one hidden layer having 16 features. The MLP accepted a total of k.l score features as inputs and returned *k* score values for all classes so that the deep learning network could be used as an aggregator for the ensemble model. Figure 5 provides the architecture of the MLP used as an ensemble to our base models.

## 4. Results and Discussion

In the present work, we used five classification measures—(i) accuracy, (ii) precision, (iii) recall, (iv) F1-score, (v) specificity to evaluate the performance of the base models and their ensemble. Since our only concern was binary classification, we have depicted all the measures as if there were two classes—(i) positive class, (ii) negative class present. Naturally, any classifier would also give the prediction class as either of the two. When the predicted class of a sample matched with its actual class then it was said to be True otherwise False. Thus we defined the five chosen classification metrics based on the terms True Positive (TP), True Negative (TN), False Positive (FP) and False Negative (FN) as follows:

1. Accuracy: It is defined as the ratio of number of correctly classified samples to that of total samples.
  (7) $$Accuracy=\frac{TP+TN}{TP+FP+TN+FN}$$
2. Precision: Precision of a class is defined as the ratio of correctly classified samples to total number of samples predicted as the given class.
  (8) $$Precision=\frac{TP}{TP+FP}$$
3. Recall: Recall of a class is defined as the ratio of correctly classified samples to total number of samples actually belonging to that class.
  (9) $$Recall=\frac{TP}{TP+FN}$$
4. F1-score: Sometimes, only Precision and Recall are not enough to measure the performance of a classifier. So, F1-score is presented to combine the both aspects as it is evaluated as the harmonic mean of the two.
  (10) $$F1-score=\frac{2\cdot Precision\cdot Recall}{Precision+Recall}$$
5. Specificity: Specificity is used to measure the proportion of negatives that are correctly identified. It is defined as the ratio of true negatives predicted to total number of samples which belong to negative class.
  (11) $$Specificity=\frac{TN}{TN+FP}$$

**Figure 6:**
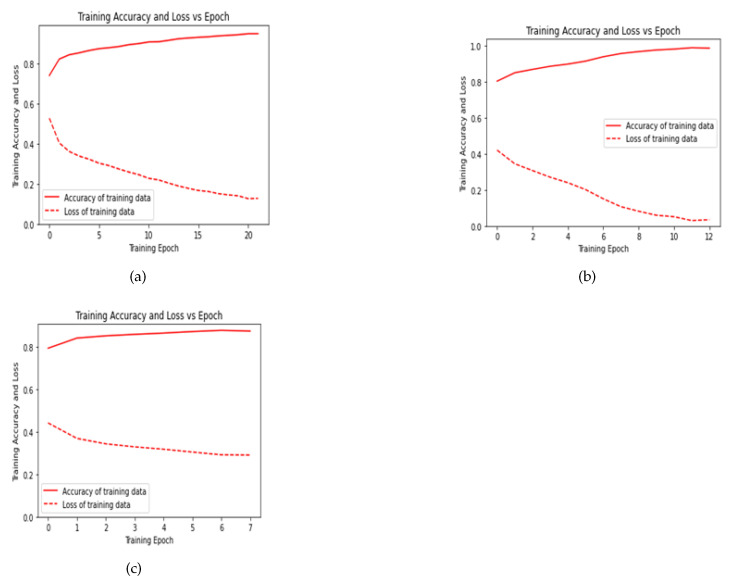
Graphical representation of training loss and accuracy vs epoch for: (**a**) Wang et al.’s [7] proposed CNN model, (**b**) Sharan et al.’s [8] proposed CNN model, (**c**) Almutairi et al.’s [10] proposed CNN-LSTM model.

Since the current problem consists of only two classes, we used binary cross-entropy as the loss function. We purposefully applied Adam optimizer to optimize the loss value throughout 100 epochs. The training procedure was performed batches with each batch having simultaneously 64 samples. We can observe the change in training accuracy and loss with epochs for all three models in Figure 6a–c.

After training, all the four classification measures were evaluated based on the test data and their prediction for each case in Table 1.

Table 1 suggests that the three chosen CNN based models were compatible for ensemble as each ensemble technique successfully increased the maximum accuracy of all three models by at least 1%. Majority voting gave least accurate results because it only considered the prediction instead of the exact probabilistic values whereas, the other three being score level fusion were able to produce somewhat better results. Trainable ensemble technique performed a little better than the non-trainable ensemble techniques probably due to the fact that weight assigning to the classification scores was performed with the help of a classifier instead of applying a pre-defined weight allocation rule. Besides, MLP itself worked as an excellent classifier because of its utilization of additional hidden features [46]. Thus, it was able to identify the patterns of classification scores as well. Among the non-trainable ensemble techniques, Choquet integral fusion worked better than sum rule because unlike sum rule, Choquet integral fusion did not assign equal weights to all three. Giving equal importance to all base models’ scores may not meet the expectations as the poor performance of an individual performance may affect the overall result. On the other hand, Choquet integral fusion assigned more weight to the model which gave more confident predictions. Among individual models, CNN-LSTM performed better than the rest two base models because LSTM considers the contexts (i.e., previous samples) along with the present sample which was beneficial for any time-series data such as, ECG signals.

We also performed experiments on the raw 1-min signal windows by taking the Table 1’s winner MLP based trainable ensemble for a performance-wise understanding between the raw data and the feature extracted data. Table 2 contains the results for raw ECG segments, which clearly shows that features extracted from the signal greatly outperformed the raw data as the final prediction made by the ensemble with raw data was only 70.77% accurate. The possible explanation for such an outcome was that classifiers may understand the pattern more efficiently in case of certain features which summarized the raw data.

Since, with this amount of data there was a possibility that the distribution of train and test sets may not be uniform, we applied two-fold cross-validation approach by swapping the train set and test set, and taking the average of both the results. The results of the base models and ensembles after this two-fold cross-validation are shown in Table 3.

We also performed five-fold cross-validation over the combined dataset of the train and test sets. The performances of the base models and ensembles after five-fold cross-validation are shown in Table 4.

From Table 4, we observe that the models and the MLP based ensemble delivered better results during five-fold cross-validation than without doing so, because in five-fold cross validation, every sample from the dataset was there in both training data and test sets at least once, and the amount of training data increased which also included some of the test data used previously. This resulted in better identification of the test data.

Furthermore, we also used standard classifiers such as SVM, ANN with a hidden layer with 100 features and Random Forest to compare how they performed with the trainable ensemble using MLP. We flattened the features for these classifiers and after prediction, reshaped the outputs for the two classes respectively before performing the ensemble on them. The results obtained by the standard classifiers and the ensemble are shown in Table 5.

Table 5 shows that the simple machine learning classifiers performed somewhat worse than the deep learning based models. So, overall ensemble was also affected by the choice of base models. Next, we compared the best performance achieved in our work for the original train and test dataset with some of the previous methods’ performance in Table 6.

From Table 6, we observe that the MLP based ensemble delivered better results than some of the previous works. Still Chang et al. [26] and Shen et al. [25] have obtained better results in their respective works. Although the ensemble worked fine for the given combination of models, their individual accuracy could not exceed 84%. So, this worked as a limiting factor for obtaining the better performance by the overall architecture. Additionally, the class-imbalance may be the reason for achieving not so high accuracy. Still, the current work held a good place among all the existing works experimented on the dataset under consideration.

## 5. Conclusions

Proper diagnosis of any severe disease such as OSA is very crucial which further emphasises the application of AI based classification techniques on such kind of data. Outcome of the competent classification models may be helpful even to the medical professionals. For example, in our case, it can help differentiating an Apnetic patient from a non-Apnetic one. In this paper, we have applied four ensemble techniques on deep learning models to detect sleep apnea based on ECG signal data. Although, each ensemble model can predict with at least 85% accurate results on PhysioNet Apnea-ECG database, still some improvements can be applied further. Since our primary focus remains on the ensemble approaches in the OSA detection domain, our experiments show that the deep learning models are compatible to be aggregated as the final ensemble method is able to surpass the best among three models. However, some modifications like considering varied LSTM based structures, attention mechanism can be thought of to the increase the base model’s performance. In future, we would consider more ensemble methods such that we can logically assign weights to fuse the classifiers’ decisions. We can also use different features from the raw data based on proper feature relevance techniques as well. From the statistics of the database used here, it is evident that the number of samples is lower than the required for any deep learning network. To overcome this problem, data augmentation techniques can be applied to produce artificial data from the original dataset.

## Figures and Tables

**Table 1:** Classification performances obtained by all the base models and their ensembles.

Model/Ensemble Technique	Accuracy (%)	Precision (%)	Recall (%)	F1-Score (%)	Specificity (%)
Wang et al.’s CNN [7]	82.36	81.54	81.78	81.65	83.93
Sharan et al.’s CNN [8]	83.29	82.32	82.80	82.53	87.39
Almutairi et al.’s CNN-LSTM [10]	84.08	83.59	82.94	83.22	86.15
Ensemble—Majority Voting	85.18	84.59	84.40	84.48	87.56
Ensemble—Sum rule	85.34	84.61	84.45	84.52	87.63
Ensemble—Choquet Integral	85.41	84.63	84.48	84.53	87.89
Trainable ensemble using MLP	85.58	84.80	84.43	84.67	88.26

**Table 2:** Classification performances obtained by considering the raw ECG segments of 1-min time-span.

Model/Ensemble Technique	Accuracy (%)	Precision (%)	Recall (%)	F1-Score (%)	Specificity (%)
Wang et al.’s CNN [7]	65.00	63.52	63.99	63.64	66.34
Sharan et al.’s CNN [8]	66.95	65.50	66.01	65.64	68.03
Almutairi et al.’s CNN-LSTM [10]	70.54	68.71	68.38	68.47	71.87
Trainable ensemble using MLP	70.77	69.38	69.96	69.57	72.19

**Table 3:** Classification performances obtained by all the base models and their ensemble using two-fold cross-validation.

Model/Ensemble Technique	Accuracy (%)	Precision (%)	Recall (%)	F1-Score (%)	Specificity (%)
Wang et al.’s CNN [7]	81.93	78.11	76.40	76.30	81.56
Sharan et al.’s CNN [8]	82.26	79.88	74.41	76.10	84.82
Almutairi et al.’s CNN-LSTM [10]	83.82	80.26	**77.79**	78.64	84.51
Trainable ensemble using MLP	84.60	82.65	76.88	79.26	86.19

**Table 4:** Classification performances obtained by all the base models and their ensemble using five-fold cross-validation.

Model/Ensemble Technique	Accuracy (%)	Precision (%)	Recall (%)	F1-Score (%)	Specificity (%)
Wang et al.’s CNN [7]	85.07	80.26	82.33	80.74	85.98
Sharan et al.’s CNN [8]	86.31	84.26	81.82	81.99	87.44
Almutairi et al.’s CNN-LSTM [10]	86.88	82.60	83.69	83.00	88.12
Trainable ensemble using MLP	87.91	84.56	84.42	84.25	88.98

**Table 5:** Classification performances obtained by standard classifiers and their ensemble.

Model/Ensemble Technique	Accuracy (%)	Precision (%)	Recall (%)	F1-Score (%)	Specificity (%)
SVM	81.64	81.33	**91.33**	**86.00**	83.65
ANN	77.48	80.85	83.29	82.01	79.17
Random Forest	78.47	78.08	90.82	83.87	79.59
Trainable ensemble using MLP	81.83	81.70	67.90	74.08	83.09

**Table 6:** Performance comparison of the previous works with our work.

Method	Features Used	Accuracy (%)	Recall (%)	Specificity (%)	Sample Window Size
Tripathi et al. [47]—Kernel Extreme Learning Machine (KELM)	EDR and HRV	76.37	78.02	74.64	-
Hassan et al. [22]—Statistical features with Extreme Learning Machine (ELM)	TQWT extracted features from Raw ECG Signal data	83.77	81.99	90.72	-
Feng et al. [48]—Feature Extraction with Deep Learning Network, SVM with Hidden Markov Model (HMM)	RRI	84.7	68.8	94.5	6000 × 1
Chang et al. [26]—1-D CNN	Raw ECG signal data	87.9	81.1	92.0	6000 × 1
Shen et al. [25]—MSDA-1DCNN	RRI then further features from MSDA-1DCNN	89.4	89.8	89.1	180 × 1
Current study—Trainable Ensemble using MLP	RRI, RAMP and EDR	85.58	84.43	88.26	240 × 3

## Data Availability

No datasets are generated during the current study. The datasets analyzed during this work are publicly available.

## Abbreviations

The following abbreviations are used in this manuscript:

OSA	Obstructive Sleep Apnea
CNN	Convolutional Neural Network
LSTM	Long Short-Term Memory
IoT	Internet of Things
SAHS	Sleep Apnea HypoApnea Syndrome
ECG	Electrocardiogram
SVM	Support Vector Machine
kNN	k-Nearest Neighbours
RF	Random Forest
RNN	Recurrent Neural Network
RQA	Recurrence Quantification Analysis
HRV	Heart Rate Variability
ANN	Artificial Neural Network
TQWT	Turnable-Q factor Wavelet Transform
AdaBoost	Adaptive Boosting
MTEO	Multilevel Teager Energy Operator
MLP	Multi-Layer Perceptron
EDR	ECG Derived Respiration
RRI	RR Interval
RAMP	R peak Amplitude
ReLU	Rectified Linear Unit
TP	True Positive
TN	True Negative
FP	False Positive
FN	False Negative
KELM	Kernel Extreme Learning Machine
ELM	Extreme Learning Machine
HMM	Hidden Markov Model
NN	Neural Network
FNN	Feedforward Neural Network
TD	Temporal Difference
RG	Residual-Gradient
MI	Mutual Information
GMM	Gaussian Mixture Model
RBF	Radial Basis Function
EMG	Electromyography
MCS	Multi-Classifier System
